# Ice nucleation in a Gram-positive bacterium isolated from precipitation depends on a polyketide synthase and non-ribosomal peptide synthetase

**DOI:** 10.1038/s41396-021-01140-4

**Published:** 2021-10-23

**Authors:** Kevin C. Failor, Haijie Liu, Marco E. Mechan Llontop, Sophie LeBlanc, Noam Eckshtain-Levi, Parul Sharma, Austin Reed, Shu Yang, Long Tian, Christopher T. Lefevre, Nicolas Menguy, Liangcheng Du, Caroline L. Monteil, Boris A. Vinatzer

**Affiliations:** 1 School of Plant and Environmental Sciences, Virginia Tech, Blacksburg, VA USA; 2 Aix-Marseille University, CNRS, CEA, UMR7265 Institute of Biosciences and Biotechnologies of Aix-Marseille, CEA Cadarache, Saint-Paul-lez-Durance, F-13108 France; 3 Sorbonne Université, Muséum National d’Histoire Naturelle, UMR CNRS 7590, IRD. Institut de Minéralogie, de Physique des Matériaux et de Cosmochimie (IMPMC), Paris, F-75005 France; 4 Department of Chemistry, University of Nebraska-Lincoln, Lincoln, NE USA; 5 Present Address: Department of Biological Sciences, University of the Sciences, Philadelphia, PA USA; 6 Present Address: Department of Microbiology and Molecular Genetics and Great Lakes Bioenergy Research Center, Michigan State University, East Lansing, MI USA

**Keywords:** Bacterial genetics, Biogeochemistry

## Abstract

Earth’s radiation budget and frequency and intensity of precipitation are influenced by aerosols with ice nucleation activity (INA), i.e., particles that catalyze the formation of ice. Some bacteria, fungi, and pollen are among the most efficient ice nucleators but the molecular basis of INA is poorly understood in most of them. *Lysinibacillus parviboronicapiens* (*Lp*) was previously identified as the first Gram-positive bacterium with INA. INA of *Lp* is associated with a secreted, nanometer-sized, non-proteinaceous macromolecule or particle. Here a combination of comparative genomics, transcriptomics, and a mutant screen showed that INA in *Lp* depends on a type I iterative polyketide synthase and a non-ribosomal peptide synthetase (PKS-NRPS). Differential filtration in combination with gradient ultracentrifugation revealed that the product of the PKS-NRPS is associated with secreted particles of a density typical of extracellular vesicles and electron microscopy showed that these particles consist in “pearl chain”-like structures not resembling any other known bacterial structures. These findings expand our knowledge of biological INA, may be a model for INA in other organisms for which the molecular basis of INA is unknown, and present another step towards unraveling the role of microbes in atmospheric processes.

## Introduction

Ice nucleation activity (INA), the ability of particles to induce the freezing of water above the freezing temperature of pure water (~−38 °C), affects many aspects of Earth’s environment. The concentration and activity of ice nucleating particles (INPs), also simply referred to as ice nuclei (IN), in mixed-phase clouds affects the ratio of frozen to liquid water in clouds because INPs trigger the freezing of super-cooled liquid water droplets and the growth of the resulting ice crystals by the Wegener–Bergeron–Findeisen (WBF) process [1]. This in turn affects how reflective clouds are, i.e., their albedo, and thus, Earth’s radiation budget and, ultimately, Earth’s temperature [2]. The WBF process also affects frequency and intensity of precipitation [3]. This is leveraged when seeding clouds to induce precipitation (or to avoid hail) [4] or when making “technical” snow in ski resorts [5]. INPs also affect at what temperature freshwater environments freeze and INPs on plant surfaces affect the severity of frost damage in crops [6]. In fact, the plant-associated bacterium *Pseudomonas syringae* was also identified as the first biological INP because it causes frost damage on corn [7]. Importantly, *P. syringae* is the most active known biogenic INP triggering freezing of water at temperatures as high as −2 °C [8]. Related species in the genera *Pseudomonas*, *Pantoea*, *Erwinia*, and *Xanthomonas* have alleles of the same *INA* gene and induce freezing at similar temperatures [9]. The *ina* gene encodes a cell wall protein that includes 50–80 repeats that are hypothesized to bind water molecules in a way to provide a scaffold for water to crystallize [10] and/or to orient water molecules to predispose them to freezing [11].

Other yet unknown genes encode INPs of yet unknown composition and structure in fungi of the genera *Mortierella* [12] and *Fusarium* [13], in lichens [14], and in plants [15]. While INA of *P. syringae* is mostly associated with intact bacterial cells, bacteria in the genera *Pantoea* and *Erwinia* can secrete the INA protein as part of vesicles [16]. *Mortierella alpina* and various *Fusarium* species, as well as birch pollen, also produce INPs that can be separated from intact cells [17, 18]. Based on differential filtration, these INPs are all in the nanometer size range, also referred to as submicron-sized INPs. Because of the different temperatures and different chemical and enzymatic treatments that inactivate INA, fungal INA may be proteinaceous [17] and INA of pollen appears to depend on polysaccharides [19]. Because most of the biological INPs are active at higher temperatures than nonbiological INPs (such as mineral dust) and some biological INPs have been found in clouds and in precipitation, biological INPs may affect Earth’s radiation budget and the intensity and frequency of precipitation [20–22]. Identifying the genes responsible for the production of biological INPs and the composition or structure of the INPs themselves is thus of key importance to determine the distribution of biological INPs in the environment and uncover their role in atmospheric processes.

We recently identified *Lysinibacillus parviboronicapiens* (*Lp*) as the first Gram-positive bacterium with INA in a freezing rain sample [9]. *Lp* is a species in the phylum Firmicutes in the order of the Bacillales [23]. INA of *Lp* (referred to as LINA from here on) is more similar to fungal INA than to the INA of *P. syringae*: LINA can be separated from bacterial cells and is resistant to proteinase K treatment and to boiling [9]. It thus appears to be secreted and not to be a protein.

Here we used comparative genomics and microbial genetics approaches that revealed a biosynthetic gene cluster, including a polyketide synthase and a non-ribosomal peptide synthetase, to be necessary for LINA. We also identified structures on transmission electron microscope images that we conclude to correspond to the LINA particles secreted by *Lp*. We discuss the possibility that other secreted, biological INPs also depend on polyketide synthases and/or non-ribosomal peptide synthetases and how this discovery may lead to a better understanding of the role of microbes in atmospheric processes.

## Materials and methods

### Bacterial strains

Eighteen *Lysinibacillus* type strains (two of which have recently been transferred to the genera *Metalysinibacillus* and *Metasolibacillus*) were obtained from three international culture collections (Table 1) and stored at −80 °C in 25% (v/v) glycerol. *Lp*, strain VT1065, had been isolated from a freezing rain sample in 2014 [8] and has been stored and maintained at −80 °C in 25% (v/v) glycerol since its initial isolation.

**Table 1:** Strains of *Lysinibacillus* species (including species recently reclassified as members of related genera), sources, INA phenotype, and accession numbers of respective genome sequences.

*Lysinibacillus* species	Original Strain ID	Source and Strain ID	Observed INA	Genome Sequence Accession #
*L. acetophenoni*	JC23	KACC 18506	-	GCF_900220965.1
*L. boronitolerans*	10a	KACC 15323	-	GCF_002200915.1
*L. chungkukjangi*	2RL3-2	KACC 16626	-	GCF_003217295.1
*L. composti*	NCCP-36	KCTC 13796	-	GCF_003856865.1
*L. fluoroglycofenilyticus*	cmg86	KCTC 33183	-	GCF_003049645.1
*L. fusiformis*	NRS 350	KACC 10903	-	GCF_003049525.1
*Metalysinibacillus jejuensis*	N2-5	KCTC 13837	-	GCF_003057615.1
*L. mangiferihumi*	M-GX18	KACC 17178	-	GCF_003049665.1
*L. massiliensis*	4400831	KACC 14317	-	GCF_002200855.1
*Metasolibacillus meyeri*	WS4626	KACC 17179	-	GCF_003049505.1
*L. odysseyi*	34hs-1	KCTC 3961	-	GCF_001591965.1
*L. pakistanensis*	NCCP-54	KCTC 13795	-	GCA_001312325.1
*L. parviboronicapiens*	BAM-582	KACC 15207	+	GCF_003049575.1
*L. parviboronicapiens*	VT1065	VT 1065	+	GCA_003049605.2
*L. sinduriensis*	BLB-1	KACC 16611	-	GCF_002200845.1
*L. sphaericus*	IAM 13420	KACC 10554	-	GCF_002982115.1
*L. tabacifolii*	K3514	KCTC 33042	-	GCF_005217585.1
*L. varians*	GY32	NBRC 109424	-	GCF_000600105.1
*L. xylanilyticus*	XDB9	KACC 15113	-	GCF_001183605.1

*KACC* Korean Agricultural Culture Collection, *KCTC* Korean Collection for Type Cultures, *NBRC* NITE Biological Resource Center, *VT* Virginia Tech.

### INA testing

Freezing nucleus spectra were determined following the droplet freezing assay protocol described previously [9]. In short, bacteria were grown at 28 °C for 48 h on Reasoner’s 2 A (R2A) plates. Six tenfold dilutions from a starting concentration of 10^8^ colony forming units (CFU) per ml were tested in triplicate using thirty 20 μL-sized droplets of each concentration. The droplets were cooled from −2 °C to −12 °C, holding for 10 min per degree. The cumulative INP concentration (*K*) per CFU was determined using the following equation [24]: *K*(*Θ*) = [ln*N*_*0*_ − ln*N*(*Θ*)]/*A*, where *N*_*0*_ equals the total number of droplets tested in one concentration, *N*(*Θ*) is the number of unfrozen droplets of that concentration at temperature *Θ*, and *A* is the average number of CFU per drop determined via dilution plating.

### Genome sequencing and assembly

A draft genome sequence of *Lp* VT1065 based on Illumina sequencing was previously published [23]. Here, the same genome was also sequenced using an Oxford Nanopore Technologies (ONT) MinION R9.4 flow cell (FLO-MIN106D). Genomic DNA was extracted using the MasterPure™ Gram Positive DNA Purification Kit, Lucigen kit. The sequencing library was prepared following the 1D Genomic DNA by ligation SQK-LSK108 protocol provided by ONT. The generated Fast5 files containing the raw signals were base-called using the ONT software Guppy 3.3.2. A hybrid genome assembly was then generated with the Unicycler v0.4.7 pipeline [25] with default parameters using the short reads from the published Illumina sequence and the long reads generated by the MinION. The assembly was annotated using Prokka 1.14.6 [26]. The assembled genome was submitted to NCBI to update the earlier submitted draft genome and has the accession number GCA_003049605.2.

### Transcriptome analysis

*Lp*, strain VT1065, was grown at 28 °C for 48 h on R2A plates, *i.e*., the same growth conditions used for INA testing. Total RNA was extracted using the hot phenol extraction method [27]. DNA contamination was removed using the Turbo DNA-Free kit (Applied Biosystems). RNA was reverse transcribed and sequenced at Novogene Inc. (Sacramento, CA) using Illumina 150 bp paired-end sequencing on the NovaSeq HiSeq 4000 sequencing platform. Raw read files were submitted to NCBI SRA with accession number SRR14385249. The downstream RNAseq analysis was performed as follows: read quality control (FastQC), sequence alignment (STAR 2.7) against the annotated *Lp* VT1065 genome, mapped read counting (FeatureCounts 2.0.1), and normalization using Transcripts Per Million (TPM).

### Comparative genomics

The genomes listed in Table 1 were annotated using Prokka 1.14.6 [26] and used as input for a pan-genome analysis with Roary 3.13.0 [28]. The analysis was run using the -e parameter to quickly generate core-gene alignments. The summary result file “gene_presence_absence.csv” generated by Roary, which consists of a matrix indicating the presence or absence of all genes in the analyzed genomes, was used to determine the genes present only in the *Lp* genomes.

### UV mutant screen

The UV mutant screen was performed following a protocol developed at the Barrick Laboratory at the University of Texas at Austin [29] with modifications and is described in detail in the Supplementary Materials and Methods S1. In short, *Lp* was exposed to UV radiation. After treatment, cells were plated onto R2A and incubated for 24–48 h at 28 °C. Suspensions of bacterial colonies were then tested for INA as described previously [9]. Genomes of mutants with reduced INA were sequenced and sequencing reads were aligned against the closed genome of *Lp* VT1065. Variant calling, including non-synonymous SNPs and single nucleotide indels were called. Identical mutations (same nucleotide substitution at the same position) that appeared in insertion sequences and genes coding for short hypothetical proteins in multiple mutants were considered sequencing errors and were not considered further. Mutations in BDABKFLJ_00841 and BDABKFLJ_00842 were confirmed by PCR and Sanger sequencing.

### Complementation of a INA-negative UV mutant

Detailed methods are provided in Supplementary Materials and Methods S1. In short, primers were designed to amplify BDABKFLJ_00841 including the 182bp-long upstream intergenic region and ending with the stop codon and restriction enzyme sites on each primer. PCR amplification was performed with a high-Fidelity DNA Polymerase using *Lp* VT1065 as template. The PCR product and the shuttle vector pHY300PLK were digested using the appropriate restriction enzymes, separated using gel electrophoresis, cut from the gel, purified, ligated together, and finally transformed into *Escherichia coli*. Colonies were checked by PCR and sequencing to ensure absence of non-synonymous mutations. A verified pHY300PLK-BDABKFLJ_00841 construct was then used to transform LINA- UV mutant BAV5288_UV14 by electroporation as described for *L. sphaericus* [30] with modifications. Single colonies were picked for further confirmation by PCR, sequencing, and INA testing.

### LINA particle purification by differential filtration

Cell-free INPs produced by *Lp* VT1065 and mutant strains 5236_UV1 and 5288_UV14 were prepared from bacteria grown on R2A plates as described previously [9]. In short, the bacterial lawn was scraped with a spatula from the agar plates after 48 h of growth at 28 °C and resuspended at an OD600 of 15.6 in water before centrifugation for 2 min at 16,000 *×* *g*. The supernatant was then filtered through a 0.22μm MillexGP Filter Unit (Merck Millipore Ltd) to remove any remaining cells. The 0.22 μm filtrate was passed through a 100 kDa Centrifugal Filter Unit (Merck Millipore Ltd) by centrifugation at 5000 × *g* for 10 min. This step was repeated about five times as only 15 mL can be processed at a time. The final retentate was resuspended in 3 mL molecular grade water (MGW) using a filter pipette and centrifuged as before (5000 × g for 10 min). This step was repeated twice more with the final retentate being resuspended with 1 mL MGW. A 100 μL sample was taken for INA testing before storing at −80 °C. A 500 μL sample from each *Lp* strain was transferred to cryo-tubes, sealed with parafilm, and shipped on dry ice to the Biosciences and Biotechnologies Institute (BIAM), Aix-Marseille Université, France for analysis by electron microscopy.

### Electron microscope imaging of LINA particles

A drop of purified LINA particle was deposited onto TEM copper grids coated with a carbon film. After 10 min, grids were stained with 1% uranyl acetate for 1 min, washed with milli-Q water and air dried. At least three grids were prepared for each the *Lp* VT1065 and the two mutants. Electron micrographs were recorded with a Tecnai G^2^ BioTWIN (FEI Company) equipped with a CCD camera (Megaview III, Olympus Soft Imaging Solutions GmbH) using an accelerating voltage of 100 kV. High-resolution TEM was carried out on a Jeol 2100F microscope, operating at 200 kV, and equipped with a Schottky emission gun, an ultra-high-resolution pole piece, and a Gatan US 4000 CCD camera. The length of the LINA filaments, the pearl-like structures, and the space between these structures were measured from TEM images using ImageJ software (v1.52p). The length of the filaments was measured on isolated filaments only, which, added to the bias of the preparation and the possibility that most filaments were broken, give an approximate length reflected by the high standard deviation obtained after the measurement of 29 filaments (488 nm ± 430 nm).

### Ultra-centrifugation of putative LINA particles

The 100 kDa retentate was spun down using an ultracentrifuge (TLS-55, Optima MAX-XP, Beckman Coulter) at 120,000 *g* for 16–18 h. The pellet was resuspended in an iodixanol solution (50% w/v in Buffer A, 200 μL) and overlaid with lower-density iodixanol solutions [50 (200 μL), 40 (400 μL), 30 (400 μL), 20 (400 μL, 10 (400 μL), and 0% (100 μL) in Buffer A] to form a step gradient (2.1 ml total) in Ultra-Clear (11 × 34 mm) ultracentrifuge tubes. The gradients were ultracentrifuged to equilibrium in a TLS55 rotor (120,000 *g*, 4 °C, 16–18 h) [31]. Ten consecutive 200 μL fractions starting from the top (fraction 1) and ending at the bottom were recovered (fraction 10) and stored at −80 °C. 10 μL of each fraction were diluted with DDW at 1:100 ratio for INA testing. Ten drops of 20 μL from each fraction were tested for INA using the droplet freezing assay described above.

## Results

### Comparative genomics and transcriptomics reveal putative LINA genes

As a first step towards identification of putative genes responsible for INA in *Lp*, we hypothesized that only *Lysinibacillus* strains with INA would contain genes responsible for INA while strains without INA would be missing these genes. We thus compared INA of one of the two *Lp* strains we previously isolated from precipitation [9] with as many strains of the genus *Lysinibacillus* as possible. We only used one of the two strains we isolated (VT1065) since genome sequencing revealed that the other strain was a clone of VT1065 (data not shown). Table 1 lists all strains we were able to obtain together with the INA results for each strain. As previously reported [23], only our *Lp* strain and the type strain of *Lp* had detectable INA under our testing conditions (see Supplementary Fig. 1).

Genome sequences of several of the tested *Lysinibacillus* strains were available publicly already. The remaining strains were sequenced to draft status by us and are now publicly available as well [23]. The genome sequence of strain VT1065 was closed complementing Illumina sequencing with nanopore sequencing on an ONT MinION^TM^. All genome sequences (Table 1) were then compared with each other to identify the genes exclusively present in *Lp*. 2993 genes were so identified as genes putatively responsible for INA in *Lp* (Supplementary Table 1).

To reduce the number of putative LINA genes further, we extracted the RNA from *Lp* strain VT1065 after being grown under the conditions that induce the strongest INA [9] and the level of expression of all genes in the genome of VT1065 was determined using RNAseq (Supplementary Table 1). 7 of the 2993 genes unique to *Lp* were found not to be expressed, reducing the number of putative LINA genes to 2986. 1385 of the 2986 genes were hypothetical while 1601 had a predicted function. Finally, since we had found LINA to be secreted and probably non-proteinaceous, we hypothesized that it may be dependent on a secondary metabolite. Therefore, the genome sequence of VT1065 was analyzed with antiSMASH [32] to identify all metabolic gene clusters (Supplementary Table 1). Among the genes that were unique to *Lp* and expressed, only 10 were identified to be core biosynthetic genes by antiSMASH.

### A UV-mutagenesis screen identifies a type I PKS–NRPS gene cluster as necessary for LINA

**Fig. 1:**
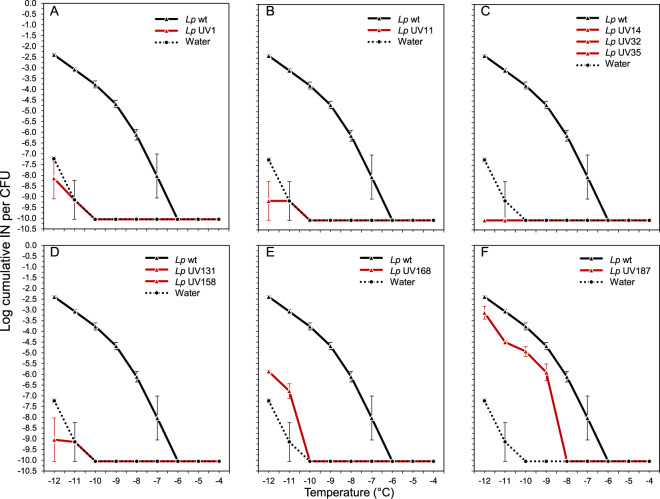
INA of *Lp* LINA-negative (or reduced) UV mutants compared to *Lp* VT1065 wild-type (*Lp* wt) based on three replicates of droplet freezing assays using 30 drops per dilution per replicate. The X-axis shows the temperature and the Y-axis shows the *log* concentration of ice nucleation particles (INP) per colony forming unit (CFU). Strains exhibiting identical spectra were combined into one graph. **A** *Lp* mutant UV1, **B** *Lp* mutant UV11, **C** *Lp* UV mutant 14, *Lp* mutant UV32, and *Lp* mutant UV35, **D** *Lp* mutant 131 and *Lp* mutant 158, **E** *Lp* mutant 168, and **F** *Lp* mutant 187.

**Fig. 2:**
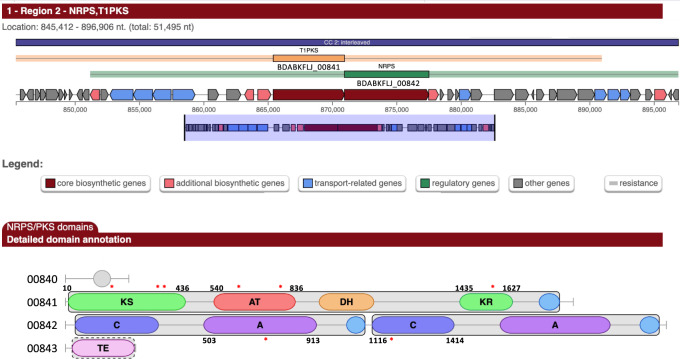
The *T1-PKS* and *NRPS* biosynthetic gene cluster necessary for ice nucleation activity (INA) in *Lp* VT1065. The graphical output obtained with antiSMASH 5.0 [32] for region 2 (corresponding to the *T1-PKS* and *NRPS* gene cluster) when using the assembled genome (NCBI Accession number GCA_003049605.2) as input is shown. Prokka annotation locus IDs were added to the region overview. The start and end positions of the domains in which mutations were identified were added to the domain annotation. The approximate locations of mutations that abolished INA are indicated by red asterisks. Precise locations are listed in Supplementary Table 2. KS: ketosynthase, AT: acyltransferase, DH: dehydratase, KR: ketoreductase, C: condensation domain, A: adenylation domain, TE: thioesterase. The 4′-phosphopantetheinylation of the carrier protein domains (acyl carrier protein domain for PKS and peptidyl carrier protein domain for NRPS) are represented by the light blue ovals.

To further reduce the list of candidate genes that may be necessary for INA in *Lp*, a UV mutant screen was performed. After UV treatment of *Lp* VT1065, seven colonies out of 18,416 colonies were found to have lost INA completely and two were found to have INA of significantly lower strength than wild-type (wt) VT1065 (Fig. 1). The genomes of all nine LINA- UV mutants were sequenced and reads were aligned against the closed genome sequence of the wild-type VT1065 strain to identify mutated genes (Supplementary Table 2). The genomes of the seven mutants that had completely lost INA, all had mutations in either one of two adjacent genes (identified as BDABKFLJ_00841 and BDABKFLJ_00842 in the Prokka genome annotation) that were unique to *Lp* based on the comparative genomics analysis and that were expressed when grown under the conditions used for INA testing. The two genes had been predicted by the program antiSMASH to encode a type I polyketide synthase (T1-PKS) and a non-ribosomal peptide synthetase (NRPS), respectively (Fig. 2). In particular, five UV LINA- strains had either one or two non-synonymous single nucleotide polymorphisms (SNPs) or a single nucleotide insertion in the *T1-PKS* gene and two LINA- strains had one non-synonymous SNP each in the *NRPS* gene each. The precise locations of the mutations within the functional domains of the two genes are shown in Fig. 2 and are listed in Supplementary Table 2. The two genes upstream of the *T1-PKS* gene were identified by antiSMASH to encode a 4′-phosphopantetheinyl transferase (BDABKFLJ_00839) and a NAD-dependent epimerase/dehydratase (BDABKFLJ_00840); the gene downstream of the NRPS gene was predicted to encode a thioesterase (BDABKFLJ_00843). Because BDABKFLJ_00843, encoding the thioesterase, is located immediately downstream of the *NRPS*, it appears to belong to the same operon as the *T1-PKS* and the *NRPS*. However, there is an intergenic region between BDABKFLJ_00840 and the *T1-PKS* gene making it unclear if this gene belongs to the same operon or not. All PKS and NRPS require 4′-phosphopantetheinylation of the carrier protein domains (acyl carrier protein domain for PKS and peptidyl carrier protein domain for NRPS), and the final product release from the PKS-NRPS assembly line requires a thioesterase [33]. The biosynthetic gene cluster can thus be expected to include the 4′-phosphopantetheinyl transferase gene (BDABKFLJ_00839) and the NAD-dependent epimerase/dehydratase gene (BDABKFLJ_00840) upstream of the PKS gene and the thioesterase gene (BDABKFLJ_00843) downstream of the NRPS gene. The five genes are likely the core of the LINA gene cluster. Flanking the five core genes are hypothetical genes that do not show homology to any known gene.

**Fig. 3:**
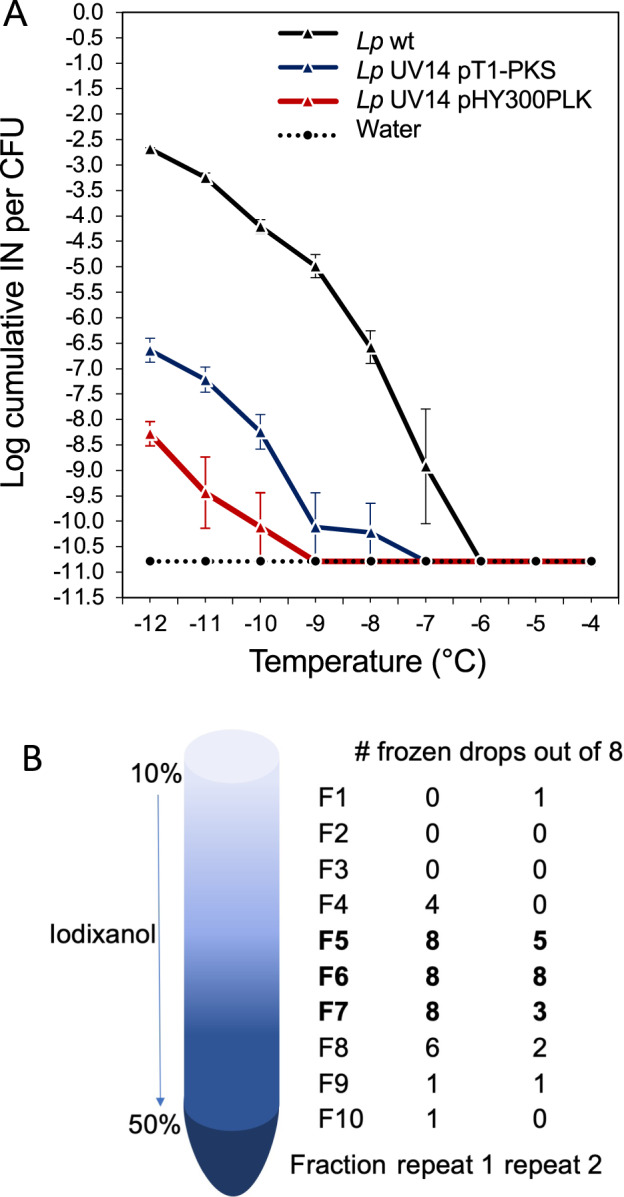
Complementation of a *T1-PKS* UV-mutant and results from gradient ultracentrifugation of the LINA particle. **A** Freezing spectra based on three replicates of droplet freezing assays (using 30 drops per dilution per replicate) of *Lp* VT1065 wild-type (*Lp* wt), *Lp* mutant UV14 with two SNPs in the *T1-PKS* gene containing the empty vector pHY300PLK, and *Lp* mutant UV14 complemented with plasmid pHY300PLK containing the *T1-PKS* gene (pT1-PKS). The X-axis shows the temperature and Y-axis shows the concentration of ice nucleation particles (INP) per colony forming unit (CFU). **B** The LINA particle was separated from *Lp* VT1065 cells and concentrated by differential filtration in a iodixanol gradient and then subjected to gradient ultracentrifugation. Samples from the indicated fractions were diluted 1:100 (to reduce the concentration of iodixanol) and analyzed by a droplet freezing assay at −11 °C (at which the difference in the number of frozen droplets was the highest). The whole experiment was repeated twice. The number of frozen droplets out of a total of 8 droplets per fraction is listed.

Since the *T1-PKS* and *NRPS* genes were unique to *Lp*, were expressed when INA was detected, were identified as core biosynthetic genes, and all UV mutants that had completely lost INA had mutations in either of the two genes, we had strong circumstantial evidence that this gene cluster was necessary for the production of the INPs produced by *Lp*. However, to confirm this conclusion, genetic complementation was attempted. Since the whole operon is ~13,000 bp long and thus challenging to clone for genetic complementation, we decided to clone BDABKFLJ_00841 together with the 182bp-long upstream intergenic region expected to contain the promoter. This region was successfully cloned into the *E. coli*—*Bacillus* shuttle vector pHY300PLK. After confirming the absence of non-synonymous mutations in the cloned gene, the LINA—UV mutant BAV5288_UV14 (carrying two non-synonymous SNPs in BDABKFLJ_00841) was transformed with the empty vector and with the resulting construct. Six independent colonies were obtained and tested for INA. Figure 3A shows that although INA was lower than in wt *Lp* VT1065, it was significantly higher than in the mutant strain BAV5288_UV14 carrying the empty vector. The INA- phenotype was thus successfully complemented and the *T1-PKS* gene was found to be necessary for LINA. While we did not complement the *NRPS* gene in any UV mutant strain, we conclude that it contributes to LINA as well since it belongs to the same biosynthetic gene cluster as the *T1-PKS* gene.

### The LINA molecule is predicted to contain a polyene chain linked to an ornithine-cysteine dipeptide

The putative LINA type I PKS is similar in sequence (40% identity, 58% similarity) with the type-I iterative PKS of *Lysobacter enzymogenes* (GenBank accession ALN58561.1), which is responsible for assembling polyene chains in the biosynthesis of HSAF (Heat-Stable Antifungal Factor), a tetramic acid-containing macrolactam [34], which is a large cyclized amide. Both the LINA PKS and the HSAF PKS have the five-domain architecture (ketosynthase–acyltransferase–dehydratase–ketoreductase–acyl carrier protein). The LINA NRPS contains two modules consisting of six domains, condensation domain 1 - adenylation domain 1 (A_1_) - peptidyl carrier protein domain 1 - condensation domain 2 - adenylation domain 2 (A_2_) - peptidyl carrier protein domain 2, and the substrate for A_1_ and A_2_ was predicted to be ornithine and cysteine, respectively. Based on this similarity in the domain organization, the LINA molecule probably contains a polyene chain linked to an ornithine-cysteine dipeptide. It is also possible that the LINA PKS could assemble two separate polyene chains, as seen in HSAF, which are condensed with the α and δ amino groups of ornithine, whereas the amino group of cysteine would condense with the carboxylate of ornithine to form the dipeptide. The nascent polyketide-peptide product is likely to be tailored by the enzymes encoded by the associated genes in the LINA PKS-NRPS cluster. Unfortunately, it is not possible to predict the precise structure of the polyketide chain from the sequence of the T1-PKS. Further investigations are needed to determine the chemical structure of the putative LINA molecule(s).

### Gradient ultracentrifugation reveals that LINA is associated with a fraction expected to correspond to vesicles

Since the polyketide linearmycin was recently found to be secreted as part of vesicles [31], we decided to test the hypothesis that the predicted LINA molecule may also be secreted as part of vesicles. We thus used the LINA particle (separated from intact cells by filtration through a 0.22 µm filter and subsequently concentrated by resuspension from a 100 kDa filter) in a gradient ultracentrifugation experiment. After centrifugation, ten fractions from the bottom to the top were each tested independently for LINA after ultracentrifugation. Figure 3B shows that only drops recovered from fractions 5, 6, and 7 had detectable INA. These correspond to the same fractions in which linearmycin had been found [31]. Therefore, this result suggests that the predicted LINA polyketide of *Lp* may be secreted as part of vesicles.

### Transmission electron microscopy shows that LINA particles are extracellular filaments composed of pearl-like nanometer-sized sub-structures

**Fig. 4:**
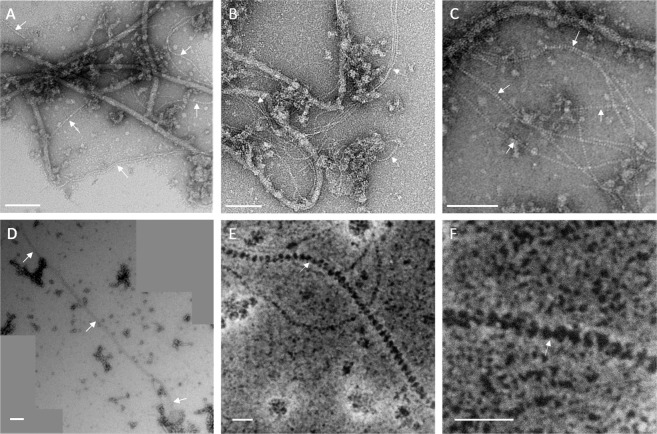
Transmission electron microscope (TEM) images of the LINA filaments excreted by the wild-type *Lp* VT1065 strain. In each panel, white arrows indicate the LINA filaments. **A**–**C** TEM images of various filaments including flagella and LINA filaments. **D** Stitching of three TEM images of long LINA filaments. **E**, **F** High-resolution images of the LINA filaments showing their “pearl chain” like structure. Scale bars represent 100 nm (**A**–**D**) and 20 nm (**E** and **F**).

Since retention of the LINA particle by the 100 kDa filter suggested a particle of a size of at least 30 nm in diameter, we hypothesized that the particle could be visualized using transmission electron microscopy (TEM). Therefore, the 100 kDa retentate obtained from wt *Lp* VT1065 (BAV4836 and BAV4837) and from INA-negative mutant strains 5288_UV14 (BAV6039) and 5236_UV1 (BAV6064) were analyzed by TEM. Several grids were observed and all wild type and mutant strains showed flagella, protein aggregates, cell debris, vesicles, and other filaments (i.e., DNA, pili) (Fig. 4A–C). But only one type structure was systematically present in the wt strain while absent from the mutants: a filament, on average 488 nm long and 5 nm wide, with a “pearl chain” structure (Fig. 4). Numerous curved filaments were observed in various sizes, likely because they were broken during the preparation as suggested by their size range between 55 nm and up to 2.2 µm (Fig. 4D). Although this could represent a bias of the preparation, the filaments were often observed aggregated together (Fig. 4B and C). They do not seem to have a rigid structure as they do not form straight lines and they are particularly curved when they are well conserved and above 1 µm in length. High-resolution (HR) TEM images revealed that each filament is composed of several connected subunits “pearl-like structures” spaced by 1.4 ± 0.2 nm, that have a particularly regular morphology being 4.8 ± 0.3 nm wide and 2.8 ± 0.3 nm thick (*n* = 90) (Fig. 4E and F). However, HR-TEM did not reveal any additional morphology of the individual pearl chain units that was not already visible with standard TEM.

## Discussion

Although the importance of ice nucleation activity for atmospheric processes and Earth’s climate has been established [2, 20, 35], the relative importance of different types of INPs in these processes and the actual physical mechanism of ice nucleation are not [36]. By isolating the ice nucleation active, Gram-positive species *Lp* from precipitation, we previously expanded the list of INA bacteria that may contribute to atmospheric processes [9]. Here we have now identified the genetic basis of INA in this new INA bacterium, which suggests that not only proteins, like the well known InaZ protein of *P. syringae* [37], but also polyketides can have INA.

*Lp* proved to be an excellent model species to study the genetic basis of INA. Comparative genomics, transcriptomics, and microbial genetics can all be used with this organism more easily than with the other INA organisms whose genetic basis of INA is still unknown, such as the fungal INA species in the genera *Fusarium* [18] and *Mortierella* [12] and plant pollen [14]. Already the genome size of the closed *Lp* VT1065 genome sequence is much smaller (~4.7 Mb) compared to *Fusarium* (~40 Mb) [38] and *Mortierella* (~38 Mb) [39] or in the plant genus *Betula* (~370 Mb) [40], the plant genus that produces pollen with the highest INA [15]. Comparative genomics and transcriptomics then reduced the already relatively small number of genes in *Lp* to only 2986 candidate LINA genes, *i.e*., genes found to be both unique to *Lp* and expressed under the conditions used to grow *Lp* before testing it for INA.

Ideally, we would have liked to use a transposon mutagenesis screen to generate LINA- mutants since transposons only cause one mutation per genome and the gene disrupted by a transposon insertion is easy to identify. However, no transposon vector for *Lp* was available to us. The UV mutant screen was a powerful alternative since next generation sequencing made it very easy to identify the mutations in the LINA- strains generated by UV treatment. The generation of multiple non-synonymous mutations or insertions/deletions in each strain (on average 4) could have made it challenging to pinpoint which of the mutations abolished LINA. However, since only two genes had non-synonymous SNPs or single nucleotide insertions in multiple LINA- strains and these genes belonged to the same biosynthetic gene cluster, the mutations in these two genes clearly stood out as the most promising candidates for being responsible for loss of LINA in the LINA- UV mutant strains. Genetic complementation of one of the two genes, the *T1-PKS*, and the fact that the two genes were among the list of expressed genes unique to *Lp*, clearly demonstrated that this gene cluster is necessary for the production of INPs by *Lp*.

This is the first time a PKS-NRPS has been found to be associated with ice nucleation. Intriguingly though, it is not the first time a polyketide synthase has been found to be associated with nucleation. There is genetic evidence for the requirement of a polyketide synthase for calcium carbonate biomineralization during otolith formation in the inner ear of Zebrafish (*Danio rerio*) [41] and Japanese medaka (*Oryzias latipes*) as well as in the formation of sea urchin spicules (*Hemicentrotus pulcherrimus*) [42]. This suggests that there may be a common mechanism between INA in *Lp* and calcium carbonate biomineralization.

The predicted structure of the individual polyketide peptide produced by *Lp* is much smaller (30–300 Da depending on the length of the tail) than the size of the INP produced by *Lp* based on its retention by the employed 100 kDa filter. Since the polyketide linearmycin was found to be secreted from *Streptomyces* cells as part of vesicles and vesicle production ceased when linearmycin production was disrupted [31], secretion of the LINA polyketide as part of a vesicle corresponding to the actual INP produced by *Lp* was a promising hypothesis. Finding LINA associated with a gradient ultracentrifugation fraction of the same density as linearmycin [31] was in fact in line with the INP vesicle hypothesis. Unfortunately, we have not been able to identify a polyketide in this fraction using liquid chromatography-mass spectrometry (data not shown). This negative result is probably due to a too low concentration of the polyketide in our preparation. However, just like for linearmycin, EM revealed that only wt *Lp* but not LINA- *Lp* mutants produce extracellular structures. Instead of vesicles, “pearl-chain”-like structures forming filaments of up to 2.2 µm were observed in WT *Lp* strains (a size in agreement with being retained by the 100 kDa filter). Such structures do not resemble any structure produced by bacteria known from the literature. These filaments produced by *Lp* could primarily serve other functions than ice nucleation, for example, they may have antibiotic activity like linearmycin [31] although a preliminary inhibition assay was negative (data not shown). Also, if INA triggered by these filaments confers a fitness advantage to *Lp* remains unknown at this point. Future studies will thus need to determine the contribution of the pearl chain structures and of INA to the fitness of *Lp*. Also, how such long filaments are formed and secreted still needs to be understood.

However, while the structure of the individual LINA polyketide peptide and the nature of the LINA INP filament needs to be investigated further, the strong genetic evidence for the dependence of LINA on the *T1-PKS* and *NRPS* genes raises the question if polyketides may also be responsible for INA in *Fusarium*, *Mortierella*, other fungi, and in pollen. INPs produced by *Fusarium*, *Mortierella*, and pollen are all secreted and in the same size range as the LINA particle [17]. While there is some indication that polysaccharides may be responsible for INA of pollen, the evidence is not conclusive [19]. INA of *Fusarium* and *Mortierella* was found to be reduced by heat and proteinase treatments but the responsible genes are unknown [12, 18]. Therefore, we conclude that polyketides and/or non-ribosomal peptides may be at the basis of, or at least contribute to, INA in these organisms as well and may be secreted as part of larger structures, as seen here for LINA INPs. Testing this hypothesis will be important to further our understanding of the yet poorly characterized submicron fraction of atmospheric INPs [43].

In conclusion, the discovery that INA of *Lp* depends on a *T1-PKS* and a *NRPS* provides a new hypothesis of the genetic basis of INA in additional organisms and should facilitate their identification. The sequences of the *Lp INA* genes and additional *INA* genes can then be used in environmental metagenomic studies to help determine their distribution. The future identification of the structure of the LINA polyketide, and possibly other INA polyketides, will also allow their direct detection in environmental samples using analytical chemistry and should facilitate the investigation of the role of biological INA in atmospheric processes.

## Supplementary information

Supplementary Methods

Supplementary Figure 1

Supplementary Table 1

Supplementary Table 2
