# Correction to: Ice nucleation in a Gram-positive bacterium isolated from precipitation depends on a polyketide synthase and non-ribosomal peptide synthetase

**DOI:** 10.1038/s41396-021-01174-8

**Published:** 2021-12-20

**Authors:** Kevin C. Failor, Haijie Liu, Marco E. Mechan Llontop, Sophie LeBlanc, Noam Eckshtain-Levi, Parul Sharma, Austin Reed, Shu Yang, Long Tian, Christopher T. Lefevre, Nicolas Menguy, Liangcheng Du, Caroline L. Monteil, Boris A. Vinatzer

**Affiliations:** 1 School of Plant and Environmental Sciences, Virginia Tech, Blacksburg, VA USA; 2 Aix-Marseille University, CNRS, CEA, UMR7265 Institute of Biosciences and Biotechnologies of Aix-Marseille, CEA Cadarache, Saint-Paul-lez-Durance, F-13108 France; 3 Sorbonne Université, Muséum National d’Histoire Naturelle, UMR CNRS 7590, IRD. Institut de Minéralogie, de Physique des Matériaux et de Cosmochimie (IMPMC), Paris, F-75005 France; 4 Department of Chemistry, University of Nebraska-Lincoln, Lincoln, NE USA; 5 Present Address: Department of Biological Sciences, University of the Sciences, Philadelphia, PA USA; 6 Present Address: Department of Microbiology and Molecular Genetics and Great Lakes Bioenergy Research Center, Michigan State University, East Lansing, MI USA

**Keywords:** Bacterial genetics, Biogeochemistry

Correction to: *The ISME Journal* 10.1038/s41396-021-01140-4, published online 23 October 2021

The middle initial was missing from the author Lefevre.

It should be Christopher T. Lefevre

The original article has been corrected.

