# Supplementary material for: Ice nucleation in a Gram-positive bacterium isolated from precipitation depends on a polyketide synthase and non-ribosomal peptide synthetase
Source: ISME J. 2021 Oct 23;16(3):890–7. doi: 10.1038/s41396-021-01140-4 (PMC8857237; doi:10.1038/s41396-021-01140-4)
Supplement: Supplementary file 1 — Supplementary Methods [file 41396_2021_1140_MOESM1_ESM.docx]

**Supplemental Methods**

Detailed methods used for the UV mutant screen and complementation of the INA-negative UV mutant are described below.

UV mutant Screen

The UV mutant screen was performed following a protocol developed at the Barrick Laboratory at the University of Texas at Austin [[1]](https://paperpile.com/c/fZ0RDn/L8jW) with modifications. Overnight cultures of *Lp* VT1065 on R2A were resuspended at an OD600 of 1.98x10^3^ and incubated for 72hr at 28°C in R2A. Bacteria were then resuspended in a 0.9% saline solution at an OD600 of 1.56. 100μL droplets were subjected to 35,000μJ of UV radiation utilizing a UV Stratalinker (Stratagene UV Stratalinker 1800). UV-treated cells were plated onto R2A and incubated for 24-48hr at 28°C. Suspensions of bacterial colonies were tested for INA at a single concentration first as described previously [[2]](https://paperpile.com/c/fZ0RDn/3TqZ). Any putative INA- mutants based on the first test were confirmed in subsequent tests and freezing nucleus spectra of each confirmed mutant were generated as described above. Confirmed mutants were sequenced using Illumina on a NovaSeq platform at Novogene Inc (Sacramento, CA). Processed reads were aligned against the closed genome of *Lp* VT1065 using HISAT2 [[3]](https://paperpile.com/c/fZ0RDn/vcDK). SAMtools [[4]](https://paperpile.com/c/fZ0RDn/jf62) was implemented to create and sort the BAM file. Variant calling, including non-synonymous SNPs and single nucleotide indels were called using VarScan [[5]](https://paperpile.com/c/fZ0RDn/V09w) with -min-coverage8 -min-var-freq 0.2 -p-value 0.05 parameters. Identical mutations (same nucleotide substitution at the same position) that appeared in insertion sequences and hypothetical proteins in multiple mutants were considered sequencing errors and were filtered out and are not shown in Supplementary Table 2. Mutations in BDABKFLJ_00841 and BDABKFLJ_00842 were confirmed by PCR and Sanger sequencing.

Complementation of a INA-negative UV mutant

Primers were designed to amplify BDABKFLJ_00841 including the 182bp-long upstream intergenic region and ending with the stop codon and restriction enzyme sites were included on each primer (Forward primer [Bgl II] AAAAAAGATCTGGTATCGAAATAGCTTCTTACTAAG, Reverse primer [Sal I] AAAAAGTCGACTTATTCAATTTGATCCCTCCCA). Genomic DNA was extracted by boiling one colony of *Lp* VT1065 for 15 minutes in 50 µL sterile water. PCR amplification was performed with Q5® High-Fidelity DNA Polymerase (NEB, Ipswich, MA, USA) according to the manufacturer’s instructions. PCR products were cleaned using the AccuPrep PCR purification kit (Bioneer, Alameda, CA, USA) and digested by the appropriate enzyme (NEB) at 37°C overnight. The shuttle vector pHY300PLK was similarly digested with the same restriction enzymes. Digested products were separated using gel electrophoresis and cleaned using AccuPrep gel extraction kit (Bioneer). Vector and PCR product were ligated using T4 DNA ligase (NEB) and transformed by heat shock into *Escherichia coli* using Alpha-Select Chemically Competent Cells (Bioline). The cloned BDABKFLJ_00841 gene was sequenced to ensure absence of non-synonymous mutations. The pHY300PLK-BDABKFLJ_00841 construct was purified with AccuPrep Plasmid mini extraction kit (Bioneer). Transformation of *Lp* with the construct was performed by electroporation as described for *L. sphaericus* [*[6]*](https://paperpile.com/c/fZ0RDn/fw0v) with modifications. Specifically, a 5-mL preculture of R2A was started from a single colony and incubated overnight at 28^o^C at 200 rounds per minute (rpm). From the preculture, two 50-mL R2A cultures were started at an OD of 0.1 and incubated at 28^o^C, 220 rpm until an OD_600_ of 0.6. Cultures were then centrifuged at 4^o^C, 4,500g for ten minutes. The supernatant was discarded and the pellet resuspended in 10mL cold double-distilled water (DDH) by pipetting up and down. The cells were centrifuged as before and resuspended in 10mL cold DDW three more times. The final pellet was resuspended in 1-mL of cold 15%-glycerol. The suspension was kept on ice while aliquoting 100μL into prechilled 1.7mL Eppendorf tubes. Aliquots were used immediately or stored at -80^o^C. 60ng (0.1-0.5μL) of plasmid were added to 100μL of cell suspension, lightly tapped to mix, then incubated on ice for 30-45 minutes. The suspension was transferred to a chilled 0.1-cm gap electroporation cuvette (Bio-Rad). Electroporation was performed using the MicroPulser (Bio-Rad) manually set to 2.0 kV. The MicroPulser contains a 10 uF capacitor and discharges an electrical pulse with an exponential decay waveform. 1mL R2A was added quickly and the suspension was transferred to an Eppendorf tube for incubation at 28°C, 200 rpm overnight. The next day, the culture was plated on LB plates containing 10 ug/mL tetracycline and incubated for two days at room temperature. Single colonies were picked for further confirmation by PCR, INA testing, and sequencing.

References

1. [Barrick Lab :: ProtocolsUVLibrary.](http://paperpile.com/b/fZ0RDn/L8jW) <https://barricklab.org/twiki/bin/view/Lab/ProtocolsUVLibrary.> [Accessed 9 Apr 2021.](http://paperpile.com/b/fZ0RDn/L8jW)

2. [Failor KC, Schmale DG 3rd, Vinatzer BA, Monteil CL. Ice nucleation active bacteria in precipitation are genetically diverse and nucleate ice by employing different mechanisms. *ISME J* 2017; **11**: 2740–2753.](http://paperpile.com/b/fZ0RDn/3TqZ)

3. [Kim D, Langmead B, Salzberg SL. HISAT: a fast spliced aligner with low memory requirements. *Nat Methods* 2015; **12**: 357–360.](http://paperpile.com/b/fZ0RDn/vcDK)

4. [Li H, Handsaker B, Wysoker A, Fennell T, Ruan J, Homer N, et al. The Sequence Alignment/Map format and SAMtools. *Bioinformatics* 2009; **25**: 2078–2079.](http://paperpile.com/b/fZ0RDn/jf62)

5. [Koboldt DC, Chen K, Wylie T, Larson DE, McLellan MD, Mardis ER, et al. VarScan: variant detection in massively parallel sequencing of individual and pooled samples. *Bioinformatics* 2009; **25**: 2283–2285.](http://paperpile.com/b/fZ0RDn/V09w)

6. [Taylor LD, Burke WF. Transformation of an entomopathic strain of *Bacillus sphaericus* by high voltage electroporation. *FEMS Microbiol Lett* 1990; **66**: 125–127.](http://paperpile.com/b/fZ0RDn/fw0v)
