# Supplementary material for: Ice nucleation in a Gram-positive bacterium isolated from precipitation depends on a polyketide synthase and non-ribosomal peptide synthetase
Source: ISME J. 2021 Oct 23;16(3):890–7. doi: 10.1038/s41396-021-01140-4 (PMC8857237; doi:10.1038/s41396-021-01140-4)
Supplement: Supplementary file 2 — Supplementary Figure 1 [file 41396_2021_1140_MOESM2_ESM.pdf]

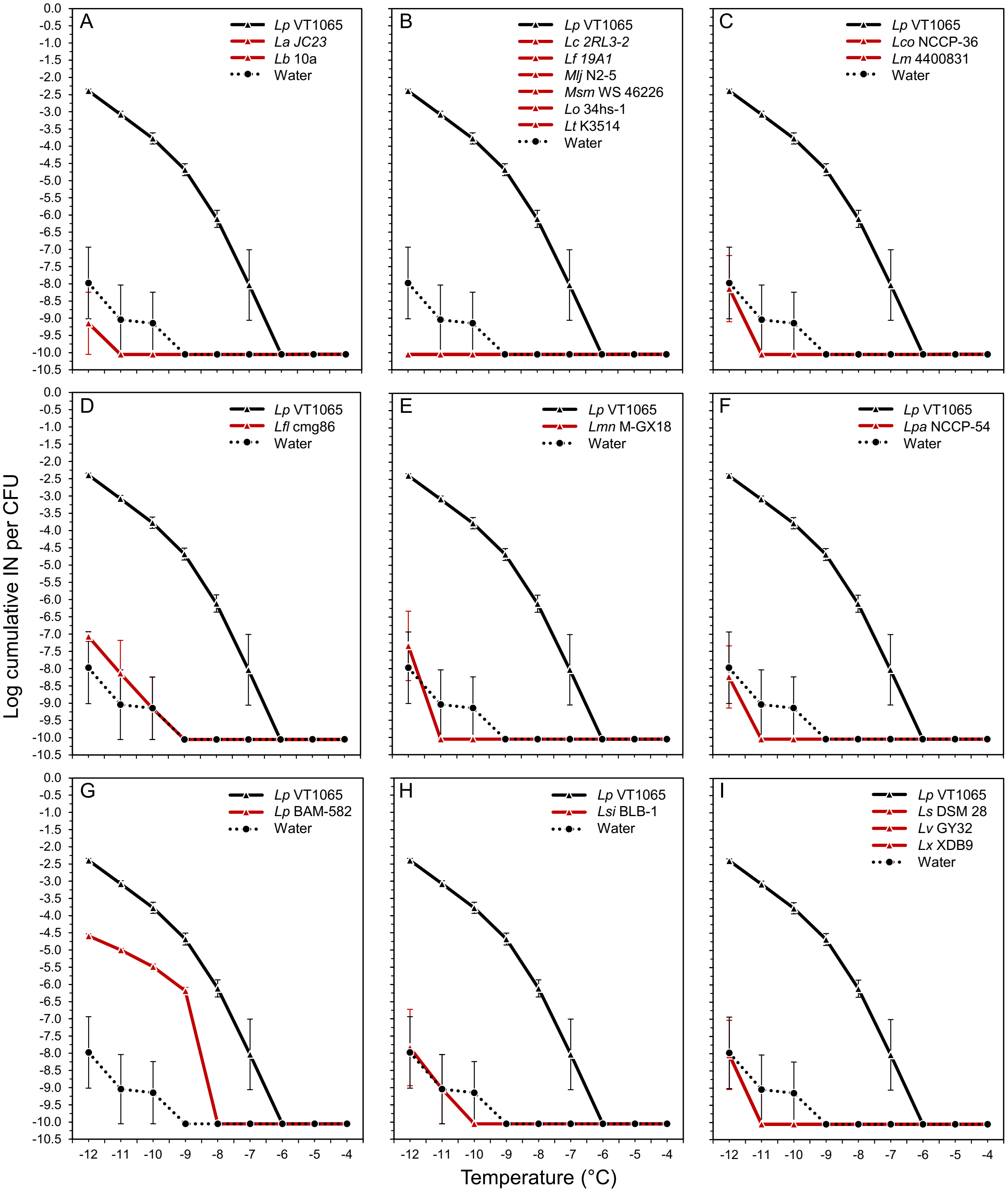

Supplementary Figure 1. Freezing spectra of *Lysinibacillus* type strains compared to *Lp* VT1065 using droplet freezing assays. The X-axis shows the temperature and Y-axis shows the concentration of ice nucleation particles (INP) per colony forming unit (CFU). Species with identical spectra have been combined in one graph. **A** *L. acetophenoni* (*La*) JC23 and *L. boronitolerans* (*Lb*) 1023, **B** *L. chungkukjangi* (*Lc*) 2RL3-2, *L. fusiformis* (*Lf*) 19A1, *L. Metalysinbacillus jejuensis* (*Mlm*) N2-5, *Metasolibacillus meyeri* (*Msm*)WS 46226, *L. odyssey* (*Lo*) 34hs-1, and *L. tabacifolii* (*Lt*) K3514, **C** *L. composti* (*Lco*) NCCP-36 and *L. massiliensis* (*Lm*) 4400831, **D** *L. fluoroglycofenilyticus* (*Lfl*) cmg86, (**E**) *L. mangiferihumi* (*Lmn*) M-GX18, (**F**) *L. pakistanensis* (*Lpa*) NCCP-54, **G** *Lp* BAM-582, **H** *L. sinduriensis* (*Lsi*) BLB-1, **I** *L. sphaericus* (*Ls*) DSM 28, *L. varians* (*Lv*) GY32, and *L. xylanilyticus* (*Lx*) XDB9. At the time the assays were performed, *Ml. jejuensis* and *Ms. meyeri* were classified as *Lysinibacillus*, though *Ml. jejuensis* did not have nomenclatural standing.
